# Genome-wide survey of tissue-specific microRNA and transcription factor regulatory networks in 12 tissues

**DOI:** 10.1038/srep05150

**Published:** 2014-06-03

**Authors:** Zhiyun Guo, Miranda Maki, Ruofan Ding, Yalan Yang, Bao zhang, Lili Xiong

**Affiliations:** 1 School of Life Sciences and Bioengineering, Southwest Jiaotong University, Chengdu, 610031, P.R. China; 2 Department of Biology, Lakehead University, Oliver Road, Thunder Bay, Ontario

## Abstract

Tissue-specific miRNAs (TS miRNA) specifically expressed in particular tissues play an important role in tissue identity, differentiation and function. However, transcription factor (TF) and TS miRNA regulatory networks across multiple tissues have not been systematically studied. Here, we manually extracted 116 TS miRNAs and systematically investigated the regulatory network of TF-TS miRNA in 12 human tissues. We identified 2,347 TF-TS miRNA regulatory relations and revealed that most TF binding sites tend to enrich close to the transcription start site of TS miRNAs. Furthermore, we found TS miRNAs were regulated widely by non-tissue specific TFs and the tissue-specific expression level of TF have a close relationship with TF-genes regulation. Finally, we describe TSmiR (http://bioeng.swjtu.edu.cn/TSmiR), a novel and web-searchable database that houses interaction maps of TF-TS miRNA in 12 tissues. Taken together, these observations provide a new suggestion to better understand the regulatory network and mechanisms of TF-TS miRNAs underlying different tissues.

Gene expression in metazoans is largely controlled by various trans-regulatory factors at various levels. At the transcriptional level, transcription factor (TF) has been considered as the primary regulator to control gene expression. By binding to specific sequences usually located in promoter regions (also known as TF binding sites, TFBS), TF can activate or repress transcription of their target genes, and form transcriptional regulatory networks^1^,^2^. In recent years, the emergence of miRNAs as another crucial suppressive regulator which share the similar regulatory logic with TFs has occurred^3^. MicroRNAs (miRNAs) are a class of short non-coding RNAs of 18 to 24 nucleotides in length that post-transcriptionally regulate various genes through direct degradation of the target mRNA and/or translational repression^4^,^5^,^6^. Abundant evidence demonstrates that miRNA control a variety of biological processes such as cell cycle, differentiation, cell proliferation, and apoptosis^7^,^8^.

Tissue-specific patterns of gene expression play fundamental roles in tissue development, distinctive features of cell types, function, and, transcriptional regulation^9^. Among the identified miRNAs, some of them exhibited tissue-specific or developmental-stage-specific expression pattern and contributed potential roles in maintaining tissue identity and function^10^,^11^. Tissue-specific miRNAs (referred to as TS miRNA) have been reported to be associated with various human diseases such as cardiovascular disease, diabetes and cancer^12^,^13^,^14^. Moreover, it has been proposed that tissue-specific gene expression patterns are controlled by combinations of TF transcriptional regulatory networks^15^,^16^. Therefore, the study of regulatory networks composed of tissue-specific miRNAs and TFs is necessary to understand tissue specificity regulation and function.

In recent years, genome-wide identification of TF-miRNA regulatory networks have been extensively studied based on the fact that TFs can regulate miRNA transcription by binding to the promoter regions of miRNA^17^,^18^,^19^,^20^. Most of these studies focus on a single tissue, or consider various tissues as a whole. However, different tissues possess a different regulatory network to perform particular functions in corresponding tissue. Therefore, systematically mapping combinatorial regulatory networks among TFs and miRNAs, especially tissue-specific miRNA and TFs across different tissues would represent a significant leap forward in disclosing molecular basis of tissue-specific gene expression, development, function and how tissue specificity is determined. In the present study, 116 experimentally validated tissue-specific miRNAs (TS miRNAs) were extracted from literatures and qRT-PCR data. It was found that half of TS miRNAs were clustered miRNAs. Here, 2,347 TF-TS miRNA regulatory relationships were identified using the TF ChIP-seq data from the ENCODE (The Encyclopedia of DNA Elements) project^21^ which provided high-resolution TFBS in multiple cell lines. Also, it was found that most TF-TS miRNA regulation occurred across multiple tissues, and most TFBS tend to enrich close to the transcription start site (TSS). Through the integration of TF expression data, we found that tissue-specific miRNAs were regulated widely by non-tissue specific TFs. In addition, 90 TF-TS miRNA regulatory relations were found which TF and TS miRNA specifically expressed in the same tissue; these regulatory relations suggest they perform their specific effect in a particular tissue. Furthermore, a series of TF-TS miRNA regulatory networks presented here, revealing that TF-gene regulatory relationships in network displayed two distinct types: type I : The highly tissue-specific or widely expressed TFs make less intensive interactions than other TFs; and type II: tissue-specific TFs participate in more interactions than other non-specific expression TFs. Finally, the TSmiR database was presented here (http://bioeng.swjtu.edu.cn/TSmiR) to provide interaction maps and expression data of transcription factor and tissue-specific miRNAs in 12 tissues. To our knowledge, this is the first systematic attempt to construct a regulatory network of tissue-specific miRNAs across multiple tissues, which can help to elucidate the molecular mechanisms of tissue-specific miRNAs network in tissue development and function.

## Results and discussion

### Identification of TS miRNAs

Using hand-curated screenings of qRT-PCR data from miRNAMap^22^ databases and literatures, 116 experimentally validated tissue-specific miRNAs from heart, skeletal muscle, lung, bone, kidney, liver, placenta, testis, brain, spleen, thymus and pancreas were retrieved. Twelve of 116 TS miRNAs (miR-1, miR-126, miR-208, miR-128a, miR-133a, miR-133b, miR-134, miR-146a, miR-377, miR-483, miR-92a and miR-95) were specifically expressed in two tissues; whereas, the remaining TS miRNAs were specifically expressed in only one tissue. After carrying out the clustering analysis for all tissue-specific miRNAs according to their genome context, half of them were found to be clustered miRNAs. Especially for the placenta, except miR-136, miR-184 and miR-381, 40 out of 43 (93.02%) placenta-specific miRNAs were clustered miRNAs, suggesting that these clustered miRNAs have similar expression patterns and play related or particular functions in this tissue. Other tissues also showed a high proportion of clustering miRNAs, for example, 7 out of 10 (70%) clustered miRNAs in brain, and 9 out of 14 (64.29%) clustered miRNAs in testis (see Table 1).

### Genome-wide identification of TF-TS miRNA regulation

Previous studies show that transcription factors, considered to regulate the transcription of microRNA, have similar mechanisms to those of protein-coding genes^23^. Therefore, the traditional approach of researching TF-gene regulatory is used to determine TF-miRNA relations. We first identified the TS miRNA TSS from high-throughput experimental data in four literature sources^11^,^24^,^25^,^26^. Next, 5 kb upstream and 1 kb downstream of each TS miRNA TSS were defined as the putative transcription factor binding region (promoter region) for each TS miRNAs based on preceding studies^27^,^28^. Previously, most studies usually produced high false positive results because position weight matrix (PWM) was used to predict the TFBS in miRNA putative prompter region^29^. Recently, ChIP-seq has emerged as a valuable and high-resolution approach to profile TFBSs *in vivo*^30^. Particularly, the Encyclopedia of DNA Elements (ENCODE) project has recently provided high-resolution TFBS in multiple cell lines to help researchers identify TF-miRNA regulation more effectively. Therefore, 2,347 TF-TS miRNA regulatory relationships were explored using highly conservative TFBS from ENCODE TF ChIP-seq data to scan the putative TS miRNA promoter region (see Supplementary Table S1).

**Figure 1:**
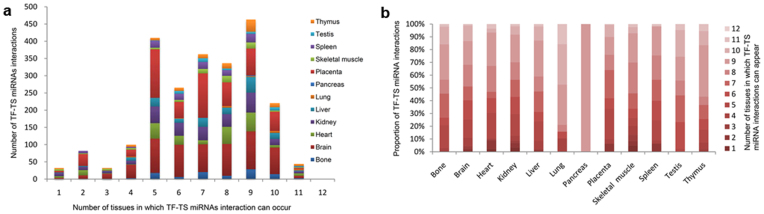
The proportion of TF-TS miRNA regulation in 12 tissues. (a) The number of TF-TS miRNA regulation distribute in 1–12 tissues in which TF-miRNA regulation can occur. (b) The proportion of TF-TS miRNA regulation. The red bar represents the number of tissues in which TF-TS miRNAs are distributed in. The deeper red indicates TF-miRNA regulatory relations have greater specificity in corresponding tissue.

To determine the distribution of 2,347 TF-TS miRNA regulatory relationships in 12 tissues, statistical analysis of the number of tissues in which TF-TS miRNA interactions can occur have been explored (see Supplementary Table S2).The result shows that most TF-TS miRNA regulatory relationships occurred in 5–9 tissues, in other words, most TS miRNAs regulated by a TF appeared in 5–9 tissues. The number of TF-TS miRNA relations decreased, accompanied by less or greater number of tissues in which TF-TS miRNA interactions can occur (Figure 1a). It suggests that TF-TS miRNA interactions which occur only in one tissue perform a specific biological function such as, tissue development and specific regulation in a particular tissue. Conversely, those TF-TS miRNA interactions which occur in the majority of tissues may possibly prefer performing a wider range of biological functions. Furthermore, the proportionate number of TF-TS miRNA interactions that occur in 1–12 tissues compared with the total TF-TS miRNA interactions of each tissue, was calculated. As shown in Figure 1b,it is apparent that non-specific distribution of regulatory relations of TF-TS miRNA are observed more in lung and pancreas tissue than other tissues, suggesting that TF-TS miRNA regulatory relationships in lung and pancreas are non-specific across 12 tissues. The largest proportion for specific distribution of TF-TS miRNA interactions is presented in placenta, this phenomenon may be related with the existence of a large number of clustered miRNAs which TF co-regulate through binding their shared promoter region.

### TF binding profiles around TSS of TS miRNA

**Figure 2:**
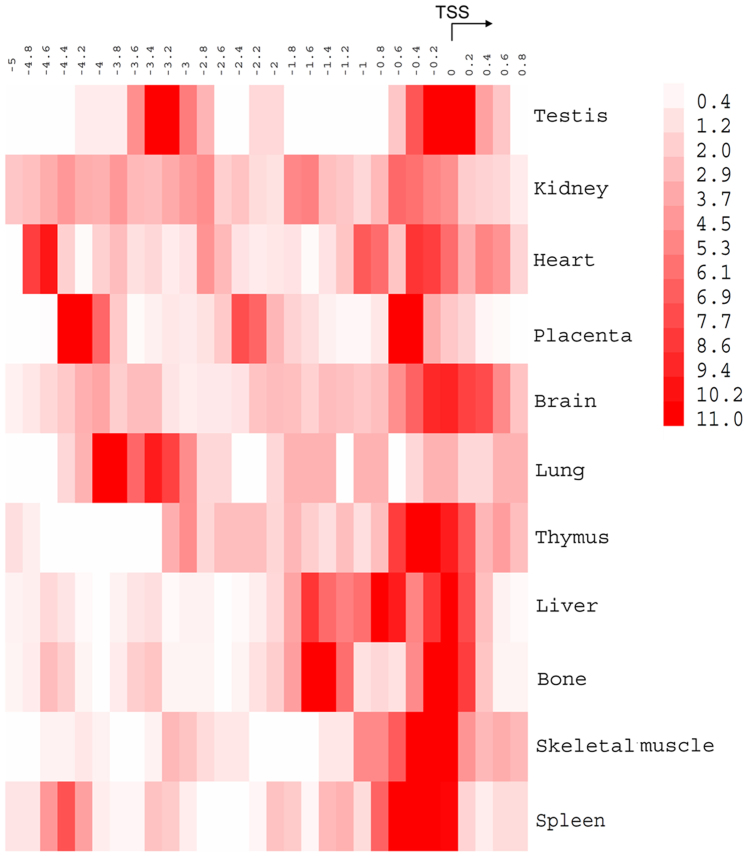
Heat map of TF binding profiles occupancy in −5 kb ~ 1 kb around the TSS of TS miRNA. The vertical color bar on right side of the heat map indicates the percent proportion of the number of TFBS overlap with each bin (200 bp) to total number of TFBS of each tissue. An arrow indicates TSS and direction of transcription initiation.

To determine TF binding profile around TSS (−5 kb ~ 1 kb) of TS miRNA, the pattern of TF occupancy was analyzed in each tissue. The number of TFs which regulate pancreas-specific miRNAs were too few, thus the TF binding profile of 11 tissues was examined (see Fig. 2). It was found that most TFBSs tend to enrich close to the TSS of TS miRNA (approximately −1 kb ~ 0.4 kb), which is consistent with previous work^31^. Significantly, for testis, heart, placenta and lung tissue-specific miRNA, there are some another enriched TFBS regions, which is located in the upstream regions of TSS of TS miRNA (−3.4 ~ −3 kb in testis, −4.8 ~ −4.4 kb in heart, −4.4 ~ −4 kb in placenta and −4 ~ −3.2 kb in lung, respectively). These TF-binding loci further away from the TSS may represent distal cis-regulatory elements for precisely regulated TS miRNA and represent involvement in tissue-specific gene expression^32^.

### The distribution of tissue-specific TFs

To determine the regulatory relationship between tissue-specific miRNA and tissue-specific TFs, the following studies were done: Firstly, the tissue-specific value (TSPV) of TFs which involved in 2,347 TF-TS miRNA regulatory relationships was calculated (see method). The lower the TSPV represents the stronger the tissue specificity. For a particular tissue, the tissue-specific value in a tissue (TSVT) determines the specific expression level of a TF; the greater the value of TSVT suggests a TF is more specific in a tissue. We set TSVT > −2.5 as threshold value to indicate a TF is tissue-specific in a tissue according to the distribution of TSVT (see Supplementary Table S1 and Fig. S1).

**Figure 3:**
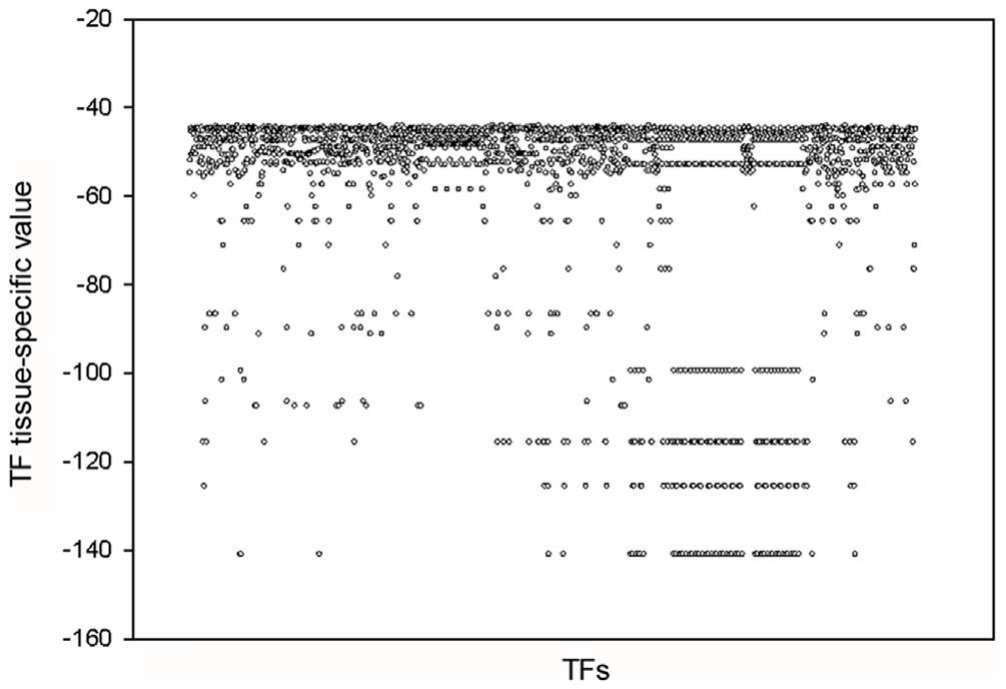
Scatterplots of TSPV of TFs. Each dot represents one TF.

**Figure 4:**
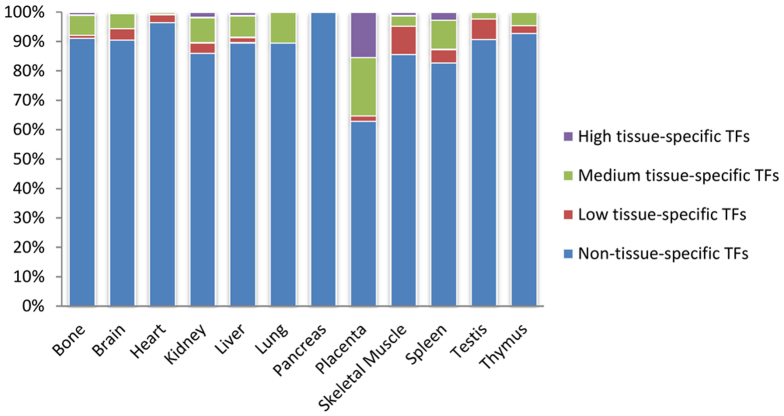
The proportion of four tissue-specific expression levels of TFs. The x-axis represents 12 tissues. The y-axis shows the proportion of the number of TFs which classed by four defined intervals to total number of TFs in each tissues.

Four tissue-specific expression levels of TFs were defined according to its TSPV: high tissue specific (tissue-specific value > = −160 and <−120, medium tissue specific (tissue-specific value > = −120 and <−80), low tissue specific (tissue-specific value > = −80 and <−60) and non-tissue-specific (tissue-specific value > = −60). For example, transcription factor CTCFL, a well-known testis-specific TF^33^, the TSPV of CTCFL is −107.2826303, is conservative according to our definition. CTCFL is a medium tissue specific TF (TSPV > = −120 and <−80), which with equivalence to the copy number of CTCFL in testis (442.6120899) is 288-fold greater than the mean copy number (1.53577) of TF in the other 11 tissues. Next, the proportionate number of TFs, classified by four tissue-specific levels, compared to the total number of TFs in each tissue was carried out. Results revealed that the majority of TFs involved in 2,347 TF-TS miRNA regulation relationships, represent non-tissue specific expression in a particular tissue, and the TSPV is mainly distributed from the range −60 to −43.02 (Fig. 3). Subsequently, the proportion of four tissue-specific expression levels of TFs across 12 tissues was explored. As shown in Figure 4, it is apparent that tissue-specific miRNAs were regulated widely by non-tissue specific TFs. MiRNAs are transcribed by RNAP II, which suggests that miRNA are regulated in a similar fashion as protein-coding genes^34^. This result was consistent with previous observations that most TF involved in tissue-specific TF-TF regulatory networks were expressed non-specifically in the corresponding tissue^35^. The finding indicates that TS miRNA perform specific functions in a tissue, such as tissue development and identity, mainly through the regulation of multiple signalling pathways instead of tissue-specific pathways. On the contrary, high or medium tissue-specific TFs suggest these TFs play a role in special functional regulation together with miRNAs in corresponding tissue.

### Tissue-specific TF-miRNA regulation

The exploration of the regulatory relationships between TFs and miRNAs both specifically expressed in the same tissue, could offer useful information to elucidate how TF-miRNA regulation plays a particular role in tissue specification or cell differentiation^36^. Therefore, the TF-miRNA regulation that is: TF and miRNA both specifically expressed in the same tissue, was screened for (Table 2). Finally, it was found that 38 TFs were involved in 90 TF-TS miRNA regulation in bone, brain, kidney, liver, lung, placenta, skeletal muscle, spleen, testis and thymus. For example, skeletal muscle-specific expression TF, serum response factor (SRF), regulates miR-1 cluster (miR-1 and miR-133) which is well known to express specifically in skeletal muscle; this signaling pathway has been certified to play a critical role in modulating skeletal muscle proliferation and differentiation^37^. The exploring of TF and TS miRNA which are specifically expressed in the same tissue will help further experimental validation studies to clarify these consistent tissue-specific TF-TS miRNA regulation and how they perform their effects in tissue specific manners.

### The identification and expression analysis of TS miRNAs target genes in 12 tissues

The function of TS miRNA is achieved mainly through the miRNA target genes at the post-transcriptional level; therefore, exploring TS miRNA-target gene pairs will help researchers to further study the regulations and functions of TS miRNA in tissue specification, physiologies, differentiation, development, etc. In order to obtain highly reliable TS miRNA target genes, the experimentally verified target genes were downloaded from miRTarBase and miRecords database. If there were no experimentally verified TS miRNA target genes, TargetScan Human was used to predict the target genes and a strict threshold filter was set to ensure the reliability of prediction (see method). Finally, 3,299 TS miRNA target genes in 12 tissues were obtained: 1,419 experimentally verified target genes and 1,880 predicted target genes. Furthermore, the functional annotation of experimentally verified TS miRNA target genes were provided using annotation tools (Supplementary Table S3).The results show that these target genes were involved in various biological processes and pathways to perform a wide range of biological functions, rather than limited to the tissue-specific functions. To determine whether TS miRNA target genes were significantly expressed specifically or not in corresponding tissue, the TS miRNA target genes were injected into the TiGER (Tissue-specific Gene Expression and Regulation) database, a comprehensive human tissue-specific gene expression database. Furthermore, the ratio of TS miRNA target genes expressed specifically in a tissue compared to the sum total number of TS miRNA target genes in a corresponding tissue, was calculated.

**Figure 5:**
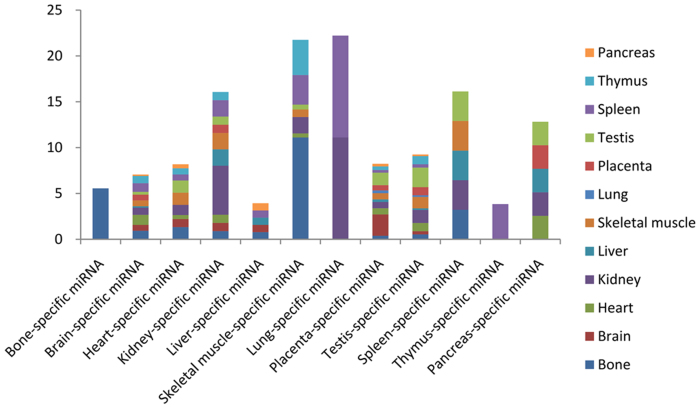
The proportion of TS miRNA target genes which were specifically expressed in 12 tissues.

The results show that most of the TS miRNA target genes specifically expressed in multiple tissues (Fig. 5). However, some TS miRNAs target genes specifically expressed in the same tissue with TS miRNA (Table 2). These TS miRNA-target gene pairs suggest that they form various networks to regulate the physiologies, differentiation, development and specification in particular tissues^38^,^39^. Significantly, bone-specific miRNA target genes only exist in bone-specific expression genes, not in the presence of non-bone tissue-specific genes. To determine if TS miRNA target genes were expressed significantly specifically in corresponding tissue, the Fisher's exact test was performed (Supplementary Table S4). It was found that kidney and testis specific miRNA target genes significantly specifically expressed in corresponding tissues (*P*-value < 0.05). However, TS miRNA target genes in other tissues did not have significant specific expression in corresponding tissues. Results suggest that most TS miRNAs are involved in different biological functions by regulating a variety of target genes in different tissues and cell types. On the contrary, the TS miRNA and their target genes which are specifically expressed in the same tissue may play an essential role in the maintenance of a specific function, such as tissue identity and differentiation in a particular tissue.

### TF-TS miRNA regulatory network

**Figure 6:**
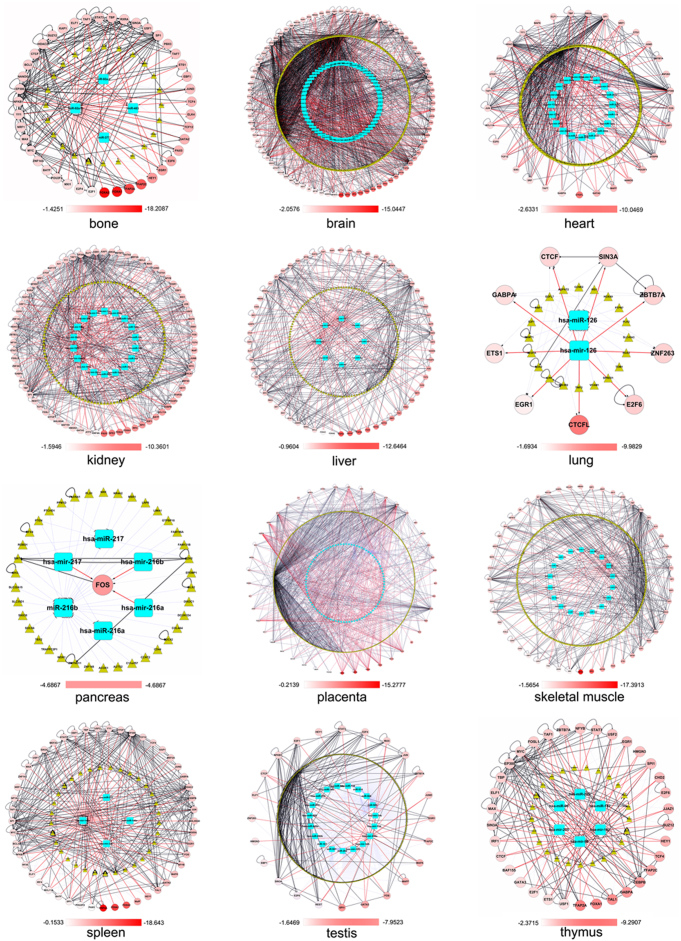
Regulatory network among TF, TS miRNA and target genes was constructed using Cytoscape. White/red color gradient circle node represents a TF; continuously numerical TSVT (where low TSVT shown in red represents a wide expression and high TSVT shown in white represents a specific expression in a tissue) are mapped to a white/red color gradient bar, circle node with minimum of TSVT beginning at the bottom (6:00) and increasing in order counter clockwise; cyan blue round rectangle node: TS miRNA; golden triangle node: target gene; TF->genes, gray dash-dot line with diamond; TF->TS miRNA, red solid line with arrow; TS miRNA-|target genes, light blue long dash line with T; protein-protein interaction: black solid line with circle.

TF and miRNA, as crucial trans-regulatory factors, have been considered to play an important role in controlling gene regulation at the transcription and posttranscriptional level. Recently, the transcriptional regulatory networks of TF-miRNA have been extensively explored; however, previous studies about TF-miRNA network did not consider the properties of tissue specificity of miRNA or expression level of transcription factors. Here, we presented a series of TF-miRNA regulatory networks that integrate verified or predicted interactions and expression data in 12 tissues and counted the number of regulatory relationships between TF, TS miRNA and target genes (see Fig. 6 and Table 3). The integrated network of 12 tissues contains 5,700 protein-protein interactions, 4,203 TF-target genes, 3,299 TS miRNAs-target genes and 2,347 TF-TS miRNAs (Table 3). In addition, to make user view network more clear, the high resolution original file (Cytoscape format, cys file) of microRNA and TF regulatory networks are provided for full exploration on our website (http://bioeng.swjtu.edu.cn/TSmiR/download.asp). Users can zoom in/out and pan for browsing the network. The networks reveal that TF-genes regulation in particular tissues showed two distinct types: type I, the highly tissue-specific or widely expressed TFs make less intensive interactions than other expression levels of TFs. For example, networks in the spleen, kidney, brain, heart, placenta, skeletal muscle. Type II: widely expressed TFs have more interactions than other TFs in the bone and liver. The regulatory network shows the different cell type will form significant molecular interactions preference and network characteristics based on the expression level of each TF. These results should prove a clue to clarify how TFs, miRNAs and target genes are coordinated to perform specific and common functions in different cell types.

### TS miRNA database

**Figure 7:**
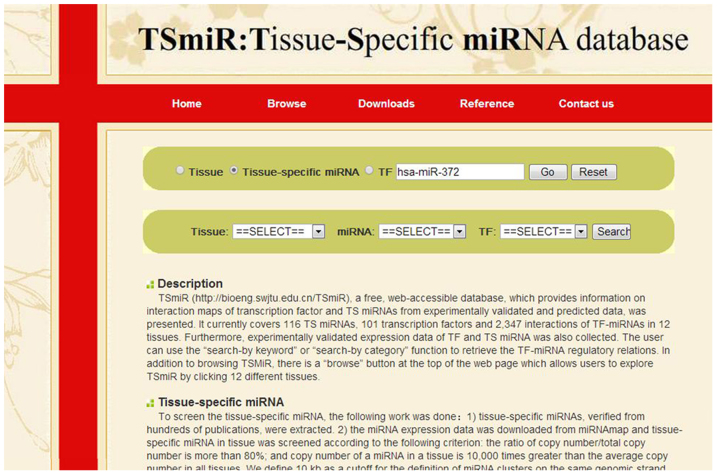
An overview of the query interface of TSmiR, which consists of “search by keyword” and “search by category” items.

Finally, here TSmiR (http://bioeng.swjtu.edu.cn/TSmiR), a free, web-accessible database, which provides information on interaction maps of transcription factor and TS miRNAs from experimentally validated and predicted data, was presented. It currently covers 116 TS miRNAs, 101 transcription factors and 2,347 TF-miRNAs regulatory relations in 12 tissues. Furthermore, experimentally validated expression data of TF and TS miRNA was also collected. The user can use the “search-by keyword” or “search-by category” function to retrieve the TF-TS miRNA regulatory relations. In addition to browsing TSmiR, there is a “browse” button at the top of the web page which allows users to explore TSmiR by clicking 12 different tissues (Fig. 7).

## Methods

### Identification of tissue-specific miRNA

To screen the tissue-specific miRNA, the following work was done: 1) the experimentally validated tissue-specific miRNAs were collected from publications^11^,^36^,^39^,^40^,^41^,^42^,^43^,^44^,^45^,^46^,^47^,^48^,^49^,^50^,^51^; 2) the miRNA expression data was downloaded from miRNAMap^22^ and tissue-specific miRNA was screened according to the copy number of miRNA in a specific tissue is 80-fold greater than the mean copy number of miRNA in other tissues. Our screening criteria is more stringent than the definition of tissue-specific miRNA (microRNA expression in a tissue is 20-fold or higher compared with the mean of microRNA expression in other tissues)^40^. According to a previous study^19^, a group of miRNAs that are consecutively located within 10 kb of distance on the same genomic strand were defined as a miRNA cluster.

### Identifying transcription start site (TSS) and promoter of TS miRNA

The TS miRNA TSS from high-throughput experimental data from four literature^11^,^24^,^25^,^26^ sources was identified: If miRNA did not have available TSS experimental information, the miRNA putative TSS was identified according to start site of each miRNA cluster. Next, the 5 kb upstream and 1 kb downstream of each TS miRNA TSS was identified as the putative transcription factor binding region (promoter region) for each TS miRNA based on previous studies^27^,^28^. At last, UCSC liftOver tool was used to convert old assembly to the current genome build (GRCh37/hg19).

### Genome-wide identification of TF-TS miRNA regulation

The highly conservative TFBS generated by ChIP-seq of ENCODE project from UCSC database was downloaded. This includes all the TFBS data extracted from the “Txn Factor Chip” track which combines TFBS from many various cell lines via the UCSC Table Browser. Thus, the TFBS were used to scan the putative miRNA promoter region to identify TF-TS miRNA regulation.

### TF binding profiles around TSS of TS miRNA

Genomic regions from 5 kb upstream to 1 kb downstream of the TS miRNA TSS were binned into 200-bp segments and the number of TFBS were calculated for each bin according to the overlap with the bin, respectively. Then, the ratio of the number of TFBS overlapped with each bin compared to total number of TFBS of each tissue was calculated. Heat maps of TF binding occupancy pattern around TSSs were generated with Cluster and TreeView software using the data produced above.

### The distribution of tissue-specific TF

The human TF quantitative RT-PCR data was downloaded from the Ravasi *et al*. study^52^. Tissue-specific values (TSPV) of 101 TFs in 12 tissues were calculated according to follow equation (1) (a simplified formula from Ravasi study^52^): 
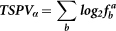
where 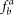 represents the ratio of expression level of TF a in tissue b to sum total expression value across 12 tissues. For 12 tissues, the smaller TSPV means that TF expresses more specifically in particular tissues; whereas, TSPV approximately equal to −43.02 (the maximum value) means TF is expressed uniformly across 12 tissues. For a particular tissue, we used the 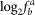 (tissue-specific value in a tissue, TSVT) to indicate which TFs are specifically expressed in this tissue. The greater the value of TSVT (maximal TSVT close to 0) suggests that TF is more specifically expressed in this tissue.

### The identification of TS miRNA target genes

Also, the experimentally verified 2,854 TS miRNA target genes from miRTarBase^53^ (release version 2.5) were downloaded, along with 5,227 validated TS miRNA target genes from miRecords^54^ (release version 3), respectively. If there was no experimentally verified TS miRNA target genes, TargetScan Human (release 6.2)^55^ were used to predict the target genes of tissue-specific miRNAs. The conserved targets were downloaded from the TargetScan and were filtered for total context score <−0.3 before further analyses. The GO term (biological process; cellular component; molecular function), Entrez gene and KEGG pathway annotation were performed using the DAVID functional annotation table tool.

### The expression analysis of TS miRNA target genes in corresponding tissue

In order to determine if TS miRNA target genes are specifically expressed in corresponding tissue, the TS miRNA target genes were injected into the TiGER^56^, a human tissue-specific gene expression database (http://bioinfo.wilmer.jhu.edu/tiger/). Firstly, for each tissue, the numbers of TS miRNA target genes specific expression in each of the 12 tissues respectively were counted, and then each of these enrichment numbers were divided by the number of TS miRNA target genes in the corresponding tissue for percentage. In order to find significant tissue-specific enriched target genes in the corresponding tissue, fisher's exact test between 12 tissues was carried out.

### TF-TS miRNA regulatory network

Finally, the 622,751 human protein-protein interaction data from BIOGRIDE^57^ database was downloaded (release version 3.2.96). Following which, the GREAT^28^ was used to predict the target genes of 101 TFs in 12 tissues, settings used as follows: Species Assembly: Human GRCh37; Gene regulatory domain: 5 kb upstream and 1 kb downstream of TSS. The network of TF, TS miRNA and target genes was constructed by cytoscape^58^ software (version 2.8.3).

## Supplementary Material

Supplementary Information Supplementary Figure S1 and Table S4

Supplementary Information Supplementary Table S1

Supplementary Information Supplementary Table S2

Supplementary Information Supplementary Table S3

## Figures and Tables

**Table 1:** Identification and cluster analysis of tissue-specific miRNAs in 12 tissues

Tissue	Mature miRNA	MiRNA clusters (<10 kb)
Heart	miR-1, miR-126, miR-208, miR-302a, miR-302b, miR-302c, miR-302d, miR-367, miR-133a, miR-133b	Cluster: (miR-302a, miR-302b, miR-302c, miR-302d, miR-367); Cluster: (miR-1-2, miR-133a-1)
Skeletal muscle	miR-1, miR-206, miR-134, miR-193a, miR-128a, miR-133a, miR-133b, miR-95, miR-208a	Cluster: (miR-133b, miR-206); Cluster: (miR-1-2, miR-133a-1)
Lung	miR-126	
Bone	miR-483, miR-377, miR-92a	
Kidney	miR-200a, miR-196a, miR-196b, miR-10a, miR-10b, miR-146a, miR-30c, miR-204	
Liver	miR-122, miR-483, miR-92a, miR-192	
Placenta	miR-377, miR-498, miR-527, miR-526a, miR-526b, miR-184, miR-154, miR-381, miR-503, miR-373, miR-371, miR-372, miR-519a, miR-519b, miR-519c, miR-519d, miR-519e, miR-516b, miR-520a, miR-520b, miR-520c, miR-520d, miR-520e, miR-520f, miR-520g, miR-520h, miR-517a, miR-517b, miR-517c, miR-450, miR-518a, miR-518b, miR-518c, miR-518d, miR-518e, miR-518f, miR-522, miR-524, miR-521, miR-523, miR-525, miR-512, miR-136	Cluster: (miR-377,miR-154); Cluster: (mir-498,mir-512-1, mir-512-2, miR-520e, miR-519e, miR-520f); Cluster: (mir-527,mir-521-1, mir-522, mir-519a-1); Cluster: (mir-526a-1, miR-523, miR-518f, miR-520b, miR-518b, miR-520c, miR-518c, miR-524, miR-517a, miR-519d); Cluster: (mir-526b, miR-519c, miR-520a, miR-519b, miR-525, miR-523, miR-518f, miR-520b, miR-518b); Cluster: (miR-503, mir-450a-1, mir-450a-2); Cluster: (mir-373, mir-371a, mir-372); Cluster: (mir-516b-1, mir-526a-2, miR-518e, miR-518a-1, miR-518a-2, miR-517c, miR-518d, miR-520h); Cluster: (mir-516b-2, miR-520d, miR-517b, miR-520g)
Testis	miR-134, miR-187, miR-34c, miR-34b, miR-507, miR-510, miR-513, miR-506, miR-508, miR-509, miR-514, miR-449a, miR-892b, miR-202	Cluster: (miR-34b, miR-34c); Cluster: (miR-506, miR-507, miR-508, mir-513a-2); Cluster: (miR-510, mir-514a-1, mir-514a-2, mir-514a-1); Cluster: (mir-509-1, mir-509-2, mir-509-3)
Brain	miR-199a, miR-199b, miR-214, miR-153, miR-137, miR-7, miR-143, miR-99b, miR-125a, miR-125b, miR-31, miR-124, miR-129, miR-138, miR-218, miR-708, miR-9, miR-128a, miR-128b, miR-186, miR-95, miR-149, miR-323, miR-330, miR-33a, miR-346, miR-93, miR-212	Cluster: (miR-199a-2, mir-214); Cluster: (miR-99b, miR-125a)
Spleen	miR-223, miR-146a	
Thymus	miR-96, miR-182, miR-205	Cluster: (miR-96, miR-182)
Pancreas	miR-216a, miR-216b, miR-217	Cluster: (miR-216a, miR-217)

**Table 2:** TF-TS miRNA regulation which TF and miRNA both specifically expressed in the same tissue^a^

Tissue	Specific expression TF-TS miRNA pairs in corresponding tissue	Specific expression TF-TS miRNAs-target genes in corresponding tissue (TF->TS miRNA-|target genes)
Bone	**E2F1/E2F4/MXI1/->miR-92a**	**miR-92a-| THBS1**
brain	TCF4->miR-199b/miR-99b/miR-125a/miR-330/mir-93NANOG->mir-199b/mir-124/mir-129/mir-9NFYB-> mir-143/mir-9/mir-330/mir-93ZBTB7A->mir-99b/mir-125a/mir-330/mir-93	miR-125b-| GRIN2AmiR-708-| GPM6AmiR-323-| PCDH8/ZNF365
heart		hsa-miR-1-|SERPINB5/TPM1
kidney	HNF4A->mir-146a/mir-30c	hsa-miR-200a-| VCAM1hsa-miR-196a/b-| HOXB8/HOXD8hsa-miR-10b-| HOXD10hsa-miR-204-| ITGB3
liver	FOXA1->mir-92a/mir-192FOXA2-> mir-92a/mir-192	hsa-miR-122-| CYP7A1
placenta	**FOSL2**->mir-527/mir-526/mir-519a/miR-516b/**mir-520d**/mir-520g/mir-520h/mir-517b/mir-517c/mir-518a/mir-518d/;mir-518e/mir-522/mir-521/mir-136**CEBPB**->mir-184/**mir-503**/mir-450/mir-136GATA2->mir-184/mir-373/mir-371/mir-372GATA3->mir-373/mir-371/mir-372HEY1->mir-503/mir-450/mir-512MAFF->mir-503/mir-450MAFK->mir-503/mir-450TFAP2C->mir-184	**miR-503-| CCNE1FOSL2->miR-520d**/e/f-| **EXPH5**miR-525-| HMOX1/ACVR2B
Skeletal muscle	**SRF->miR-1**/mir-133aEGR1-> mir-193aFOS-> miR-1/mir-133aHSF1-> mir-193aUSF2-> mir-193a	**SRF->miR-1-| TPM1/TPM2**
testis	REST->mir-134/mir-449aE2F6->mir-449aEBF1->mir-449aHMGN3->mir-449aSIN3A->mir-449a	miR-506-| MYB/BCAT1/RFX4/SNX16/CHODL/APH1B/DMRT1miR-202-| AGBL2/RAG1/BOLL/C6orf204/SCML4
Lung	ETS1->miR-126EGR1-> miR-126	
thymus	USF1-> mir-96/mir-182	
Spleen	BATF->mir-223BCL11A->mir-146aELF1->mir-146aIRF4->mir-146aPAX5->mir-146aPOU2F2->mir-146a/mir-223RFX5->mir-146aSPI1->mir-146a	

aWord in bold represent factor which involved in TF->TS-miRNA-|target gene regulation all specifically expressed in the same tissue.

**Table 3:** Statistics of the number of interactions between TF, TS miRNA and target genes

Interactions	Bone	Brain	Heart	Kideny	Liver	Lung	Pancreas	Placenta	Skeletal muscle	Spleen	Testis	Thymus
Protein-protein interactions	150	1763	631	522	337	14	11	1043	457	275	389	108
Edges TF->target genes	111	1176	438	433	177	24	3	1166	166	220	82	207
Edges TS miRNAs->target genes	18	637	452	112	127	18	39	1032	245	31	562	26
Edges TF->TS miRNA	101	630	226	277	163	19	3	583	83	110	43	109
