# Supplementary material for: Genome-wide survey of tissue-specific microRNA and transcription factor regulatory networks in 12 tissues
Source: Sci Rep. 2014 Jun 3;4:5150. doi: 10.1038/srep05150 (PMC5381490; doi:10.1038/srep05150)
Supplement: Supplementary Information — Supplementary Figure S1 and Table S4 [file srep05150-s1.pdf]

Genome-wide survey of tissue-specific microRNA and transcription factor regulatory networks in 12 tissues

Zhiyun Guo[1], Miranda Maki[2], Ruofan Ding[1], Yalan Yang[1], Bao zhang[1],and Lili Xiong[1]*

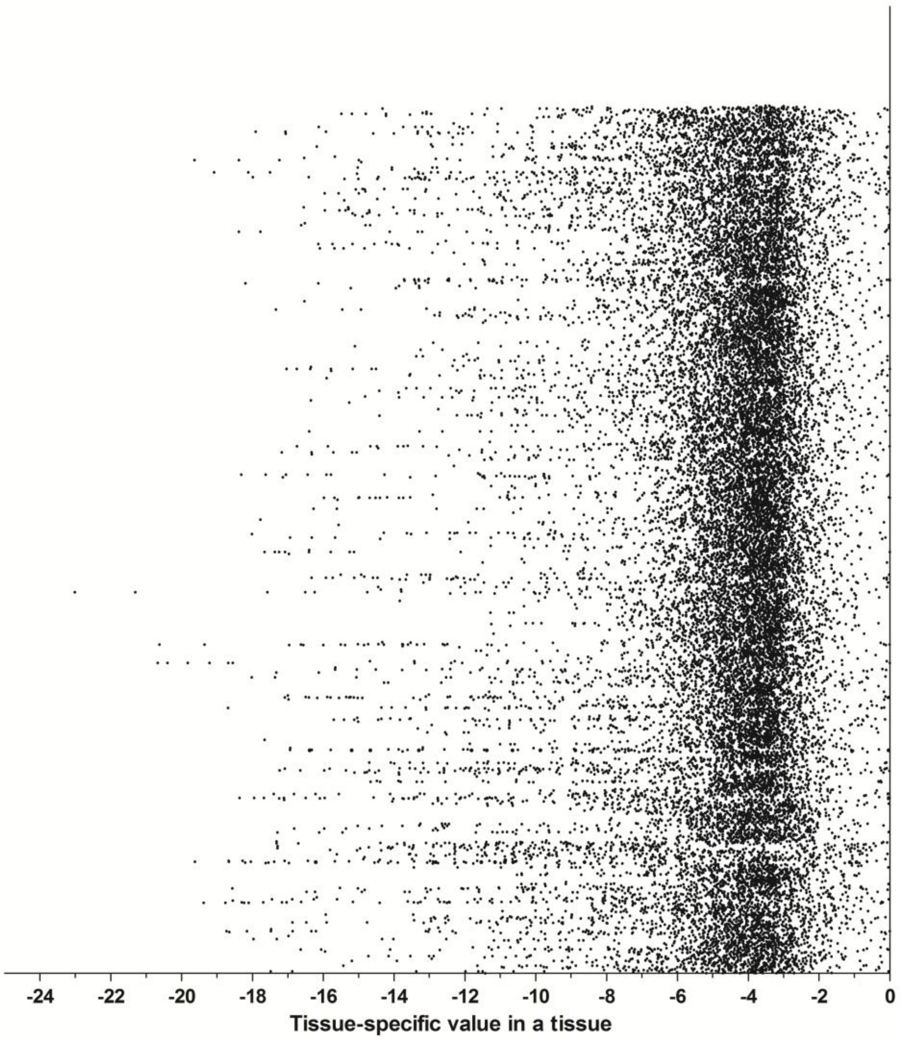

Fig. S1 A graph of scatter plot that shows the distribution of TSPV of TFs. Each dot represents one TF. The greater TSPV suggests a TF is more specific in a tissue.

Table S4 Fisher's exact test

	Bone specific?		Brain specific ?		Heart specific ?		Kidney specific		Liver specific ?		Lung specific ?		Pancrea sspecific ?		Placenta specific ?		Skeletal _muscle specific ?		Spleen specific ?		Testis specific ?		Thymus specific ?	
	Y	N	Y	N	Y	N	Y	N	Y	N	Y	N	Y	N	Y	N	Y	N	Y	N	Y	N	Y	N
The enrichment No. of target genes of corresponding tissue-specific miRNAs	1	27	4	32	2	27	6	33	1	9	0	4	0	8	6	9	2	33	0	22	12	28	0	25
The enrichment No. of target genes of another 11 tissues miRNAs	17	3254	633	2630	450	2820	106	3154	126	3163	18	3277	39	3252	1026	2258	243	3021	31	3246	550	2709	26	3248
P-value(2-Tail)	0.14257708451774134		0.28785450377495836		0.4169082234955823		0.0017491416928738651		0.3250509635137603		1		1		0.577021685036164		1		1		0.03476431619114987		1	
